# The EGFR-GEP100-Arf6-AMAP1 Signaling Pathway Specific to Breast Cancer Invasion and Metastasis^†^

**DOI:** 10.1111/j.1600-0854.2009.00917.x

**Published:** 2009-04-24

**Authors:** Hisataka Sabe, Shigeru Hashimoto, Masaki Morishige, Eiji Ogawa, Ari Hashimoto, Jin-Min Nam, Koichi Miura, Hajime Yano, Yasuhito Onodera

**Affiliations:** 1 Department of Molecular Biology, Osaka Bioscience InstituteOsaka 565-0874, Japan; 2 Graduate School of Biosciences, Kyoto UniversityKyoto 606-8607, Japan; 3 Department of Neurosurgery, School of Medicine, Oita UniversityOita 879-5593, Japan; 4 Department of Thoracic Surgery, Faculty of Medicine, Kyoto UniversityKyoto 606-8507, Japan; 5 Laboratory of Diagnostic Pathology, Kyoto University HospitalKyoto 606-8501, Japan

**Keywords:** AMAP1, Arf6, breast cancer, EGFR, GEP100, invasion, metastasis, membrane traffic, molecular target, tumor microenvironment

## Abstract

Tumors are tissue-specific diseases, and their mechanisms of invasion and metastasis are highly diverse. In breast cancer, biomarkers that specifically correlate with the invasive phenotypes have not been clearly identified. A small GTPase Arf6 primarily regulates recycling of plasma membrane components. We have shown that Arf6 and its effector AMAP1 (DDEF1, DEF1, ASAP1 and centaurin β4) are abnormally overexpressed in some breast cancers and used for their invasion and metastasis. Overexpression of these proteins is independent of the transcriptional upregulation of their genes, and occurs only in highly malignant breast cancer cells. We recently identified GEP100 (BRAG2) to be responsible for the Arf6 activation to induce invasion and metastasis, by directly binding to ligand-activated epidermal growth factor receptor (EGFR). A series of our studies revealed that for activation of the invasion pathway of EGFR, it is prerequisite that Arf6 and AMAP1 both are highly overexpressed, and that EGFR is activated by ligands. Pathological analyses indicate that a significant large population of human ductal cancers may utilize the EGFR-GEP100-Arf6-AMAP1 pathway for their malignancy. Microenvironments have been highly implicated in the malignancy of mammary tumors. Our results reveal an aspect of the precise molecular mechanisms of some breast cancers, in which full invasiveness is not acquired just by intracellular alterations of cancer cells, but extracellular factors from microenvironments may also be necessary. Possible translation of our knowledge to cancer therapeutics will also be discussed.

Development of tumor malignancy, including the acquisition of abnormal invasive and metastatic activities, is a consequence of the accumulation of genetic mutations in tumor cells. However, in the case of mammary tumors, biomarkers that specifically correlate with their invasive phenotypes have not been clearly identified, in spite of extensive research including those on genomic mutations and gene expression (1–4). Accordingly, the molecular machineries specifically involved in the invasion of breast tumor cells have not been clearly understood. On the other hand, Liu et al. (5) have recently proposed a 186-gene ‘invasiveness’ gene signature. This signature, however, appears to rather represent a signature of oncogenic transformation, and may not directly mediate invasion (6).

The Arf family of small GTPases regulate membrane trafficking and remodeling (7). Arf GTPases are conserved throughout eukaryotic evolution (http://smart.embl-heidelberg.de/smart/do_annotation.pl?DOMAIN=ARF&BLAST=DUMMY&EVOLUTION=Show#Evolution). Interestingly, Arf GTPases are found in early eukaryotes such as *Giardia lamblia*, in which no members of the Ras family of GTPases are found (8). This fact implies that control of membrane traffic and remodeling by Arf GTPases is more essential for some eukaryotic life than the control of proliferation and life cycle by Ras GTPases. There are six isoforms of Arf GTPases in mammals (Arf1-6), although Arf2 has been lost in humans. Arfs are classified into three classes by structural similarity: class I (Arf1-3), class II (Arf4 and 5) and class III (Arf6). Class I and class II Arfs primarily function at the Golgi, and are involved in intracellular secretory processes (9). On the other hand, Arf6 is the most divergent of the Arf isoforms, and primarily functions at cell peripheries by regulating endocytosis and recycling-back of plasma membrane components, as well as several types of cell surface receptors (10). Several functions of Arf6 are closely linked to the functions of Rac, and Arf6 has been shown to play pivotal roles also in actin-cytoskeletal remodeling at the cell periphery (10–12). Arf6 has moreover been shown to play crucial roles in higher orders of various cellular functions (13–15), including Fcγ-receptor-mediated phagocytosis (16), disassembly of E-cadherin-mediated epithelial cell–cell adhesions (17,18), recycling of integrin β1 (19), and in tumor invasion and metastasis (20,21).

Arf6 is ubiquitously expressed in different tissues and organs in adults. We found that Arf6 and its effector AMAP1 are abnormally overexpressed in malignant breast cancer cells, and this Arf6 signaling pathway is specifically involved in the invasive and metastatic activities of some breast cancer cells. Pathological analyses also revealed that several components of this Arf6 pathway are excellent indicators of the invasive and malignant phenotypes of primary breast cancers. In this article, we discuss Arf6 signaling in cancer invasion and metastasis, together with the molecular mechanisms by which this Arf6 pathway functions (Figure 1). We also discuss the possible translation of our knowledge to cancer therapeutics.

**Figure 1:**
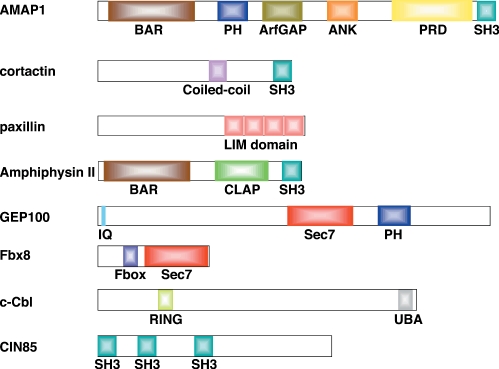
Schematic representation of proteins discussed in this review.

## Arf6 is Abnormally Overexpressed Only in Highly Invasive Breast Cancer Cells

Invasive characters are diverse even among the different cell lines of breast tumors (22,23). On the other hand, a direct correlation between *in vivo* invasive phenotypes and *in vitro* invasion activities has been demonstrated in a number of different breast cancer cell lines (24,25). We have shown that the Arf6 protein is overexpressed in all of the highly invasive breast cancer cell lines we examined, to levels 10- to 20-fold higher than those observed in noninvasive breast cancer cell lines as well as human normal mammary epithelial cells (HMECs) (20). We also found that Arf6 is predominantly localized to invadopodia and plays an essential role in invadopodia formation as well as other invasion activities of different breast cancer cells, including MDA-MB-231 (20). Our results suggested that Arf6 might be a predictive biomarker for the invasive phenotypes of primary cancers of the human breast. However, in spite of our extensive efforts, we have not succeeded in generating an anti-Arf6 antibody, which is applicable for immunohistochemistry of clinical specimens. Besides breast cancer, Arf6 has also been shown to be a component of invadopodia of a melanoma cell line LOX, although it has not been investigated as to whether Arf6 is overexpressed in melanomas (21).

## Posttranscriptional Upregulation of Arf6 Protein Expression

**Figure 2:**
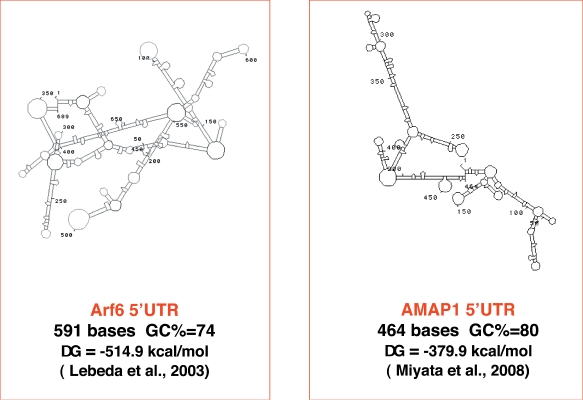
The secondary structure of the 5’-untranslated regions of Arf6 mRNA and AMAP1 mRNA.

Gene expression profiling analyses have not identified the *Arf6* gene as being overexpressed in invasive ductal carcinomas (IDCs). Our analysis on cultured breast cancer cell lines has instead revealed that all of the breast cancer cell lines we examined express comparable levels of *Arf6* mRNA, regardless of their invasiveness (20). HMECs also expressed high levels of *Arf6* mRNA, as observed in highly invasive breast cancer cell lines. Thus, the selective overexpression of the Arf6 protein in highly invasive breast cancer cells appears to be independent of the enhanced transcription of the *Arf6* gene, but may be because of its posttranscriptional regulation. Consistent with this notion, the 5’-untranslated region of the *Arf6* mRNA is very long and possesses a very complicated secondary structure with a very high level of free-energy change [http://www.ncbi.nlm.nih.gov/entrez/viewer.fcgi? view=graph&val=NT_026437. 11&_gene=ARF6] (Figure 2). mTOR is one of the central kinases regulating 5’-cap-dependent translational control (26), and is frequently overexpressed in breast tumors (27). We however have preliminary results indicating that *Arf6* mRNA is not under the control of mTOR (OBI, Osaka).

## Suppressive Control of the Arf6 Activity by Non-Canonical Ubiquitination, and its Dysfunction in Breast Cancer Cells

Several small GTPases, such as H-Ras, RhoA and Rac1, have been shown to undergo ubiquitination (28–30). Arf6 is also ubiquitinated (31). In the database there is a protein named Fbx8, which contains a Sec7 (ArfGEF) domain and an F-box domain (32). Members of F-box proteins act as subunits of the Skp-Cullin-F-box (SCF) complexes of ubiquitin E3 ligases, and primarily determine substrate specificity of the ubiquitination through their direct interaction with substrates (33). Fbx8 was found to form a complex with Cullin1 through its binding to Skp1, and mediate the ubiquitination of Arf6, but not other Arf isoforms (31). This ubiquitination of Arf6 occurs predominantly in the polymer form, which is however not linked to the immediate proteasomal degradation. It has been shown that RhoA and Rac1 are polyubiquitinated, which is linked to their proteasomal degradation, while ubiquitination of H-Ras occurs in mono- or di-ubiquitination forms and is not linked to the proteasomal degradation (28–30). We have shown that the ubiquitination of Arf6 interferes with its interaction with guanine nucleotide exchange factors (GEFs) and GAPs, and hence inhibits Arf6 from functioning (31). Therefore, Fbx8-mediated ubiquitination provides a novel suppressive mechanism of the Arf6 activity, not known with other small GTPases. Moreover, protein expression of Fbx8 is impaired in many breast cancer cell lines, while its forced expression inhibits invasive activities (31). Therefore, impaired expression of Fbx8 appears to be one of the factors contributing to the invasive phenotypes of some breast cancers.

## AMAP1 is an Effector for GTP-Arf6 in Invasion and Metastasis

Active forms of the Ras superfamily of small GTPases generally interact with other proteins, called effectors, to transmit their downstream signals. AMAP1 (also called DDEF1 in human, DEF1 in bovine, and ASAP1 or centaurin β4 in mouse) has an ArfGAP domain and was originally reported to exhibit efficient GTPase-activating protein (GAP) activities against Arf1 and Arf5, but very weak GAP activity against Arf6 (34,35). On the other hand, we have provided several lines of evidence that AMAP1 acts as an effector for Arf6 in tumor invasion, in addition to its function as a GAP for other Arf isoforms. Cellular Arf6 recruits AMAP1 to sites of its activation (both the plasma membrane and the cytoplasmic large vesicles), when activated such as by epidermal growth factor (EGF) (36). AMAP1 co-localizes with Arf6 at invadopodia, and its knockdown effectively blocks invadopodia formation and invasive activities (37).

Biochemically, AMAP1, via its ArfGAP domain, binds directly and stably to GTP-Arf6 without immediate hydrolysis even in the presence of divalent cations, while this domain exhibits only marginal levels of the stable binding to GDP-Arf6 nor to Arf1 and Arf5, regardless of their nucleotide binding status (36). All of these properties are also shared with AMAP2/PAG3/Pap α close isoform of AMAP1 (38). We have already discussed why ArfGAPs like AMAP1 and AMAP2 should have such complicated properties, exhibiting enzymatic GAP activities to the class I and II Arfs on one hand and binding to the class III Arf (i.e. Arf6) on the other (14,39).

## AMAP1 is an Indicator of the Malignant Potentials of Breast Cancer

Like Arf6, AMAP1 is also abnormally overexpressed (> 10 fold) in highly invasive breast cancer cell lines, as compared with weakly- and noninvasive breast cancer cell lines and HMECs (37). Immunohistochemical analysis also revealed that AMAP1 protein expression is very high in all of the IDCs, *n* = 19, while its expression is at basal levels in most ductal carcinoma *in situ* (DCIS; six out of seven cases) which is noninvasive, and in noncancerous components. However, AMAP1 protein expression was high in all cases of DCIS in patients simultaneously bearing IDC lesions (*n* = 9). Therefore, AMAP1 protein expression appears to be well correlated with the malignant potentials of primary cancers of the human breast, rather than being simply correlated with the invasive phenotypes (37).

Like Arf6, overexpression of the AMAP1 protein in highly invasive breast cancer cells is also independent of the transcriptional upregulation of the *AMAP1* gene (37). The 5’-UTR of *AMAP1* mRNA also possesses a very complicated secondary structure with a high free-energy change ((40), Figure 2). We have observed that AMAP1 protein levels are downregulated in serum-starved MDA-MB-231 cells, which is then upregulated swiftly upon EGF stimulation; and moreover, rapamycin reduces the AMAP1 protein levels in MDA-MB-231 cells (OBI, Osaka). The 5’-UTR of *AMAP1* mRNA exhibits an internal ribosomal entry site (IRES) activity in differentiated monocytes (40). However, we have yet to study the precise mechanism involved in enhanced translation of the *AMAP1* mRNA in malignant breast cancer cells (40).

## Overexpression of AMAP1/DDEF1/ASAP1 in Other Types of Tumors

Amplification of chromosome 8q is frequently observed in a broad range of solid tumors, including breast cancer (41). The *AMAP1/ASAP1/DDEF1* gene is located at 8q24.12 (42,43). The DDEF1 protein has been shown to also be overexpressed in high-grade primary uveal melanomas, in which amplification of chromosome 8q was found to correlate most strongly with the expression of the *DDEF1* mRNA (42). The ASAP1 protein is also overexpressed in 80% of primary prostate cancers, in which its expression is substantially higher in metastatic lesions; and moreover, additional copies of its gene are detected in 58% of primary prostate cancer specimens (44). Like breast cancer cells, siRNA-mediated knockdown of ASAP1 protein expression in prostate cancer cells has been shown to effectively block their migration and invasion (44). Thus, the genetic alterations and mechanisms involved in AMAP1/ASAP1/DDEF1 overexpression differ among different types of cancers. We have shown previously that AMAP1 also appears to be involved in the invasion of glioblastomas and lung cancers (45). It waits to be investigated as to how widely AMAP1 is involved in the invasion and metastasis of tumors with different organ and tissue origins.

## GEP100 Activates Arf6 to Induce Invasion and Metastasis

The human genome encodes 15 different proteins bearing the Sec7 domain (the GEF domain for Arfs), which is three times more than the number of Arf GTPases (7,^15^,46). Among them, GEP100 (also called BRAG2) is responsible for Arf6 activation which induces breast cancer cell invasion and metastasis (47). GEP100 has been well-documented biochemically to be a specific GEF for Arf6 (48). On the other hand, other GEFs, including ARNO and EFA6 which are also known to be robust GEFs against Arf6, are not immediately involved in the invasive activities, while these GEFs are also expressed in breast cancer cells (47). Overexpression of GEP100 together with Arf6 made otherwise noninvasive MCF7 cells to be highly invasive, whereas co-overexpression of ARNO and Arf6 did not (47). Therefore, distinct types of GEFs activate Arf6 and evoke different functions of Arf6 even within a single cell. Most ArfGEFs bear distinct multifactorial protein- and lipid-interaction modules (13). Different protein binding and/or membrane environments of each GEF might determine their different involvement in distinct cellular functions of Arf6. Such differences among the Arf6GEFs might also be closely related to the different ARF6 cargo specificities (also see later).

Most ArfGAPs also have several multifactorial protein interaction modules (39), and the above notion for ArfGEFs might also be true for ArfGAPs. On the other hand, one should be very careful because forced overexpression of different GEFs for Arf6 often evokes similar phenotypes of plasma membrane blebbing and protrusion, which are prototypes of Arf6 activation. The same is also true for the overexpression of ArfGAPs, which might cause shut down of the general activities of cellular Arf6.

## EGFR Activates GEP100 through Direct Interaction

**Figure 3:**
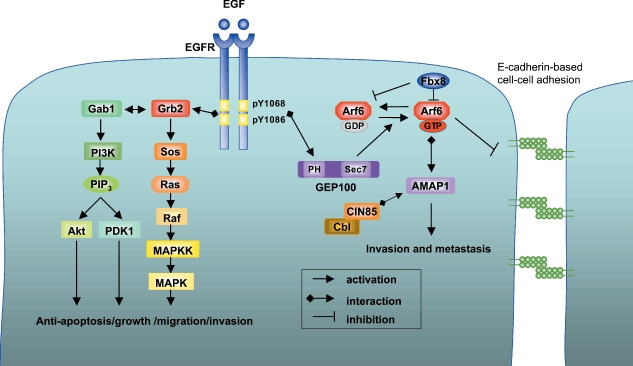
The EGFR-GEP100-Arf6-AMAP1 pathway specific to cancer invasion and metastasis Besides well-known EGFR signaling pathways that link to the cell invasion and migration via activation of Ser/Thr kinases, EGFR can evoke a novel pathway that leads to the cancer invasion and metastasis, in which phosphorylated Tyr1068 or Tyr1086 binds directly to the PH domain of GEP100. Through this binding, GEP100 activates Arf6 and its downstream signaling pathway employing AMAP1 as its effector. For functioning of this pathway, it is prerequisite that both Arf6 and AMAP1 are highly overexpressed, and that Tyr1068 or Tyr1086 is highly phosphorylated. This EGFR-GEP100-Arf6-AMAP1 pathway plays pivotal roles not only in the formation and function of invadopodia, but also in perturbation of the E-cadherin-based cell–cell adhesion. Arf6 can be ubiquitinated by Fbx8, a component of the SCF complex. Loss of Fbx8 expression is frequently observed in different breast cancer cells, and contributes to their invasiveness. Monoubiquitination of AMAP1 by Cbl, via AMAP1's binding to CIN85, is also necessary for the invasion.

Epidermal growth factor receptor (EGFR) is frequently overexpressed in a large number of cancers, including breast, lung, prostate, head and neck, and colon, and is a biomarker of the poor prognosis of many of these diseases (49,50). Moreover, EGF stimulation has been shown to evoke invasive activities in some breast cancer cells, including MDA-MB-231 (51). We have shown that GEP100 directly binds to ligand-activated EGFR to activate Arf6 (47). This binding is mediated via an interaction of the pleckstrin homology (PH) domain of GEP100 either with the Tyr1068- or Tyr1086-phosphorylation sites of EGFR (47). The human genome contains almost 100 different PH domains; and only about 15% of them have been shown to bind specifically to phosphoinositides, while most PH domains may exhibit some detectable affinities to phosphoinositides (52). This is the first demonstration that a PH domain binds to phosphotyrosine. Consistently, both Tyr1068 and Tyr1086 become heavily phosphorylated in breast cancer cells upon EGF stimulation (47). On the other hand, EGFR has nine major tyrosine phosphorylation sites (53). Phosphorylation of Tyr1173 is very weak, and phosphorylation of Tyr992, Tyr1045 and Tyr1148 is almost undetectable in EGF-treated MDA-MB-231 cells (47). Phosphorylation of Tyr845 has also been reported in MDA-MB-231 cells (54). Phosphorylation of Tyr1068 and Tyr1086 are both known to provide binding sites for the src homology 2 (SH2) domains of Grb2, a binding which may then lead to activation of the Ras-MAPK pathway. However, the EGFR-GEP100 pathway does not necessarily interfere with the EGF-dependent proliferation of breast cancer cells (47); see Figure 3.

## Mechanisms by Which the Arf6 Pathway Functions in Tumor Invasion (I): Roles in Invadopodia Formation and Phagocytosis

**Figure 4:**
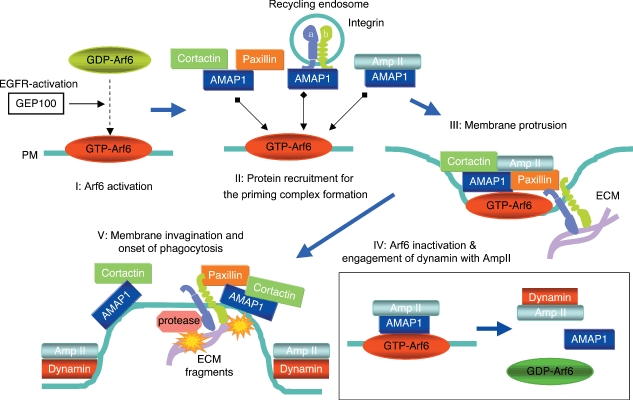
Hypothetical model as to how the EGFR-GEP100-Arf6-AMAP1 pathway functions in tumor invasion Formation and function of invadopodia consist of several steps, I–V, as depicted in this figure. I: Arf6 is activated by GEP100, bound to the ligand-activated EGFR, and resides at or near the plasma membrane (PM). II: GTP-Arf6 then recruits its effector AMAP1, which is associated with several different proteins including cortactin, paxillin, and perhaps also amphiphysin II (Amp II). AMAP1 is also indirectly associated with certain types of integrins, and acts to recruit them to the plasma membrane. III: AMAP1, as well as its accessary proteins, then remodel plasma membrane and cytoskeletal architecture to form membrane protrusions, which contain integrins. IV: Inactivation of Arf6 is thought to precede the onset of the phagocytosis at invadopodia, although this is not yet completely proofed. An ArfGAP responsible for the Arf6 inactivation in invadopodia is not identified. At around the same time, dynamin should be recruited to invadopodia, a recruitment that is presumably mediated by amphyphisin or some equivalent molecules. For the recruitment of dynamin by amphyphisin, amphyphisin needs to release the prebound AMAP1. V: Invadopodia are sites of the phagocytosis of degraded extracellular matrices, which bind to integrins. The Bar domain of AMAP1 is thought to play a crucial role for invagination of the plasma membrane. Dynamin is also an integral component for phagocytosis, which mediates the fission reaction of the endocytic vesicles from the plasma membrane. A cue to start phagocytosis is not yet clarified. Ubiquitination of AMAP1 by Cbl is not described in this figure, for simplification.

Tumor cells invade into basement membranes *in vitro* through specialized structures, called invadopodia. Bowden et al. have shown that cortactin and paxillin, both known to be involved in actin-cytoskeletal remodeling, are integral components of the invadopodia of MDA-MB-231 cells (22). We have shown that AMAP1 bridges cortactin and paxillin, in which a proline-rich sequence of AMAP1 binds to the src homology 3 (SH3) domain of cortactin, and the SH3 domain of AMAP1 binds to paxillin (37). This trimeric protein complex is clearly detected only in highly invasive breast cancer cells in which AMAP1 is abnormally overexpressed, but not in noninvasive breast cancer cells or normal mammary epithelial cells (37). Paxillin and cortactin are relatively abundant proteins in most cultured cells, including both invasive and noninvasive breast cancer cells, and levels of the AMAP1 expression seem to be crucial for formation of this trimeric protein complex at detectable levels. Blocking of this trimeric protein complex inhibits invadopodia formation as well as metastatic activities of breast cancer cells (37; also see later). On the other hand, it should be noted that AMAP1 may have an alternative way of the complex formation with cortactin. The SH3 domain of AMAP1 is known to also bind to Fak (55). Bharti et al. have reported that binding of the AMAP1 SH3 domain to Fak may mediate a complex formation of AMAP1 with cortactin in NIH 3T3 cells (56). On the other hand, by expressing the AMAP1 SH3 domain, we have shown that the AMAP1 SH3 domain is predominantly used for the binding to paxillin, but not to cortactin, in malignant breast cancer cells ((37); Figure 4).

Invadopodia are sites of the plasma membrane protrusion. Fcγ receptor-mediated phagocytic cups of macrophages are also formed as a consequence of the plasma membrane protrusion; and it has been shown that Arf6 is localized to Fcγ receptor-mediated phagosomes and are necessary for their formation (16). The basic function of GTP-Arf6 includes the ‘outward flow’ of membrane components and proteins into the plasma membrane (10,^57^,58). Consistently, Arf6 activated by EGF stimulation of cells, as well as Arf6Q67L, recruit AMAP1 to the plasma membrane (36). It is thus plausible to assume that GTP-Arf6 might act to recruit AMAP1 and its binding partners, including cortactin and paxillin, to the plasma membrane; and these proteins then start to remodel the plasma membrane, as well as the cytoskeleton, to form protrusive structures.

It has been shown in a mouse model of human breast cancer that the β1 integrin subunit is pivotal for the tumor progression, while its absence results in a state of tumor cell dormancy (59). Moreover, α3β1 integrin is highly implicated in invasion and metastasis of many primary breast cancers, as well as MDA-MB-231 cells (60,61). Our preliminary results indicate that AMAP1 also makes a complex with β1 integrin to recruit it to sites of the Arf6 activation (OBI, Osaka).

Invadopodia are also sites of matrix degradation and phagocytosis of the degraded matrix fragments by use of integrins. Fcγ receptor-mediated phagocytic cups become invaginated during phagocytosis of opsonized particles. We have shown that AMAP2 (also called Papα and PAG3), a close isoform of AMAP1, is localized to Fcγ receptor-mediated phagocytic cups as its integral component (62). AMAP1 and AMAP2 both interact with several endocytic proteins, including amphiphysin IIm and intersectin, to recruit them to sites of the Arf6 activation (39,62). Moreover, like amphiphysin II (63), AMAP1 and AMAP2 each has a Bar domain, which together with the PH and ArfGAP domains, efficiently bend the surface of large unilamellar vesicles (64). An electron microscopic observation has indeed shown that AMAP2, as well as Arf6, are localized at invaginated pits of the plasma membrane (38).

AMAP1 is ubiquitinated, and this ubiquitination also appears to be necessary for the involvement of AMAP1 in invasion (65). A mouse ortholog of AMAP1, ASAP1, has been shown to bind to CIN85 (66), which is an interacting protein of Cbl, an E3 ligase (67). AMAP1 was then found to form a complex with Cbl through its binding to CIN85 (65). Cbl mediates ubiquitination of AMAP1 mostly as monoubiquitination, and this ubiquitination is not immediately linked to the rapid proteasomal degradation (65). Cbl also mediates monoubiquitination of ligand-activated EGFR at multiple sites (68,69), and this ubiquitination has been shown to be closely linked to recruitment of the ligand-activated EGFR to endocytic pathways and their lysosomal degradation to downregulate the EGFR signaling (70,71). Given that AMAP1 forms a complex with integrins, it is possible that monoubiquitination of AMAP1 is necessary for its lysosomal recruitment, in order to degrade the matrix fragments phagocytosed by AMAP1-bound integrins. On the contrary, however, interaction of AMAP1 with CIN85 has been implicated in recycle-back of EGFR to the plasma membrane (72). Understanding the role of AMAP1 monoubiquitination, as well as its interaction with CIN85, is an aspect important to clarify the precise roles of AMAP1 in invasion.

Dynamin is a large GTPase that mediates fission reaction of vesicles from the donor membrane, and is hence important for the final onset of the phagocytosis (73). Dynamin interacts with endocytic proteins including amphiphysin IIm, and locates at the neck of budding vesicles (73). Our results implicated that change of the binding partner of amphiphysin IIm from AMAP2 to dynamin-2 is necessary for the final onset of endocytosis (36,38). On the other hand, there is an implication that hydrolysis of GTP bound to Arf6 is also necessary for endocytosis of certain receptors, such as transferrin receptors (74). It waits to be clarified whether hydrolysis of GTP bound to Arf6, as well as what kind of other cues (*ex*, cues for the changing of the partner of amphiphysin IIm from AMAP1/2 to dynamin-2) are necessary for the final onset of phagocytosis from invadopodia.

## Mechanism by which the Arf6 Pathway Functions in Tumor Invasion (II): Roles in the Disruption of E-cadherin-based Cell–Cell Adhesion

In addition to the formation of invadopodia, disruption of E-cadherin-based cell–cell adhesion is a hallmark of the acquisition of invasive and metastatic phenotypes of most tumors with epithelial origins (75–77). Arf6 activity is thought to be pivotal for the assembly and disassembly of E-cadherin-based cell–cell adhesions: it has been shown that an active form of Arf6, Arf6Q67L, causes disassembly of E-cadherin-mediated adherens junctions in MDCK cells, while its inactive form, Arf6T27N, blocks the hepatocyte growth factor (HGF)-induced internalization of E-cadherin-based junctional components (17,18). Experiments using fetal hepatocyte cells prepared from *Arf6*–/– mice also support this notion (78). We have shown that the expression and activation of Arf6 by GEP100 in MCF7 cells by cDNA transfection perturbs their E-cadherin-based cell–cell adhesion (47). Consistently, GEP100 has been previously implicated in endocytosis of E-cadherin (79) and recycling of β1 integrin (80), although the precise mechanisms are unknown. We have shown, on the other hand, that the activation of Arf6 by other GEFs, such as ARNO, does not block E-cadherin-based cell–cell adhesion in MCF7 cells (47).

Cadherins and integrins do not possess conventional endocytic signals. Endocytosis of such plasma membrane proteins devoid of conventional endocytic signals (hence thought to be endocytosed independent of clathrin) has actually been implicated to occur along a pathway regulated by Arf6 (81–83). In the case of MHC-I, which also lacks a conventional endocytic signal, Arf6 has been shown to regulate the formation of EHD1-containing tubules, which then regulate MHC-I recycling (84). On the other hand, recent reports have shown that E-cadherin can be endocytosed through the clathrin-dependent pathway (17,^18^,85); and that GTP-Arf6 is also involved in the recruitment of the clathrin/AP-2 complex to membranes (86,87). Molecular mechanisms as to how Arf6 is involved in endocytosis of E-cadherin are totally unknown.

## The EGFR-GEP100-Arf6-AMAP1 Pathway in Primary Tumors of the Human Breast

About 20–40% of breast tumors have been reported to be positive for EGFR (88). We observed that about 40% of ductal carcinoma *in situ* (DCIS) specimens are positive for EGFR, while EGFR positivity *per se* does not correlate with their grades or comedo forms (the most aggressive form) (47). On the other hand, about 60% of DCIS specimens were positive for GEP100, while GEP100 positivity *per se* did not correlate with tumor grades (47). However, we found that double-positivity for EGFR and GEP100 expression correlated with tumor grades (47). Moreover, more than 80% of EGFR-positive DCIS of grades 2 and 3 were positive for GEP100 (47). In the case of IDC, about 40% were also positive for EGFR and more than 80% were positive for GEP100 (47). The difference in GEP100 positivity between IDC and DCIS specimens was also found to be statistically significant. Again, more than 90% of EGFR-positive IDC specimens were positive for GEP100 (47). These observations are supportive of the notion that EGFR predominantly utilizes GEP100 for the development of malignancy in mammary ductal carcinomas. These observations, together with the aforementioned observations of Arf6 and AMAP1 expression, also suggest that the EGFR-GEP100-Arf6-AMAP1 pathway is frequently used in a substantially large population of primary ductal carcinomas for the development of malignancy.

Among the four members of the EGFR family, ErbB2 (also called Neu and Her2) is also overexpressed in 20–30% of breast tumors, while the coexpression analysis did not find a significant positive or negative correlation between EGFR and ErbB2 overexpression (89–91). About 90% of the comedo forms of DCIS are positive for ErbB2, and DCIS stratified according to Scott's system showed that ErbB2 is related to a subgroup with worse prognosis (90). Fifteen to thirty percent of IDCs also show *c-erbB2* gene amplifications and its overexpression (90). We have shown that co-overexpression of GEP100 and Arf6 in MCF7 breast cancer cells induces invasiveness, which is substantially dependent on EGF stimulation (47). On the other hand, overexpression of ErbB2 is sufficient to induce the migration and invasion of MCF7 cells, even in the absence of ligands (92). Induction of the malignant phenotypes by ErbB2, however, appears to be mediated at least partly through its pleiotropic functions, including transcriptional inactivation of the *E-cadherin* gene (93), direct interaction with the E-cadherin protein to perturb E-cadherin function (94), and association with the laminin receptor α6β4 integrin (95). We have previously shown that ErbB2, expressed at a basal level in MDA-MB-231 cells, is not coprecipitated with GEP100 (47). However, these results do not deny the possibility that ErbB2 can associate with GEP100, when ErbB2 is overexpressed at very high levels by its gene amplification and hence heavily tyrosine phosphorylated (see below). Moreover, EGFR can heterodimerize with ErbB2 to produce a more robust signal than EGFR–EGFR homodimers (89,93). ErbB2 contains a tyrosine residue which may correspond to Tyr1068 and Tyr1086 of EGFR (50). Therefore, it will be worth investigating whether GEP100 also has the ability to interact with ErbB2, that is highly overexpressed and heavily tyrosine phosphorylated. If this is the case, cancer cells, bearing overexpressed ErbB2 and the GEP100-Arf6-AMAP1 pathway, may not require exogenous factors for their invasion and metastasis. It will also be interesting to examine whether the EGFR/EGFR homodimer or the EGFR/ErbB2 heterodimer, when activated by ligands other than EGF (89), employ GEP100. Moreover, it also waits to be determined whether other members of the EGFR family, besides EGFR and ErbB2, also employ GEP100.

Hepatocyte growth factor receptor (HGFR; also called c-Met) tyrosine kinase is also expressed in 20–40% of breast tumors and is highly implicated in cell motility and malignancy (96,97). HGFR also contains tyrosine residues which are heavily phosphorylated upon activation. If GEP100 also interacts with HGFR besides EGFR, about 40–80% of breast cancers may utilize the GEP100-Arf6-AMAP1 pathway, although this might not be the sole pathway for their invasion and malignancy.

## Fight Against Breast Cancer by Use of Knowledge Regarding the Arf6 Pathway in Invasion

The EGFR-GEP100-Arf6-AMAP1 pathway provides several novel molecular targets for breast cancer therapeutics. As already discussed (98–100), this pathway might provide alternative targets for the treatment of patients with cancer that has become resistant to the currently available EGFR inhibitors. Moreover, overexpression and amplification of EGFR in breast cancers correlates inversely with their estrogen receptor status (101–103). Therefore, this pathway may also provide alternative targets for patients with cancer resistant to endocrine treatments.

siRNA-mediated knockdown of GEP100, Arf6 and AMAP1 each effectively inhibits the invasive activities of breast cancer cells (20,^37^,47). However, Arf6 is expressed ubiquitously in different types of cells, organs and tissues, and may have housekeeping roles. AMAP1 and GEP100 are also expressed widely. Therefore, general knockdown of their expression might cause some unexpected side effects to human health. On the other hand, only highly invasive breast cancer cells exhibit detectable levels of the complex formation of AMAP1 with cortactin (37). This binding interface appears to provide an excellent molecular target (45). In this complex, AMAP1 binds to the SH3 domain of cortactin via its proline-rich sequence, SKKRPPPPPPGHKRT (P4 peptide, (37)). There are almost 250 different SH3 domains encoded in the human genome (104), and SH3 domains bind to their cognate proline-rich ligands generally with a one-to-one stoichiometry (105). On the other hand, the AMAP1 and cortactin binding is very atypical in its stoichiometry, in which one AMAP1 P4 peptide binds to two cortactin SH3 domains simultaneously (45). In accordance with this, fine structural analysis has revealed a very unusual way of binding of SH3-Pro in the AMAP1-cortactin complex (45). Therefore, it should be possible to design specific blockers of this SH3-Pro binding that do not effectively inhibit other canonical SH3/Pro bindings. Indeed, we have demonstrated that a cell permeable form of the P4 peptide, as well as the small compound UCS15A, effectively block AMAP1/cortactin binding and block breast cancer invasion and metastasis, while these blockers do not efficiently inhibit other canonical SH3/Pro bindings (45).

The binding interface between EGFR and GEP100 also appears to be unusual, given that PH domains generally exhibit affinities to phosphoinositides (52). Moreover, a small molecule inhibitor that specifically interacts with the Sec7 domain of cytohesins has been reported (106). Generation of small molecule inhibitors that specifically block the EGFR and GEP100 binding, or interact with the GEP100 Sec7 domain will be useful for cancer therapeutics.

## Future Perspectives

Extensive efforts are being carried out to develop gene expression profiling-based diagnostic tests for breast tumors, including those for prognostic tests and predictive tests for endocrine- and chemotherapy-sensitivity. It should be kept in mind, however, that recent studies using comparative genomic and proteomic profiling have documented a lack of correlation between the mRNA and protein levels of numerous genes (107,108). mRNAs under posttranscriptional control encode proteins involved in cell adhesion, signal transduction, growth control, and transcriptional control, hence posttranscriptional control of protein production provides rapid and specific responses, such as to stress, apoptosis, and proliferative and oncogenic stimuli. Accordingly, our study revealed that abnormally high levels of expression of Arf6 and AMAP1 proteins in malignant breast cancer cells are primarily because of the posttranscriptional regulation of their mRNAs or proteins. Further development of ‘protein expression profiling’ technology will definitely contribute to the more fine stratification of patients.

Our results show that the EGFR-GEP100-Arf6-AMAP1 pathway requires EGF for activation. It has been highly implicated that tumor-associated macrophages, rather than carcinoma cells, are the major source of EGF in mammary tumors (109,110). Consistently, over 80% of cases of human breast cancers bearing high densities of macrophage accumulation in their microenvironments have a poor prognosis (111). The crucial roles of macrophages in the development of the invasive and metastatic phenotypes of mammary tumors have also been documented in a mouse model experiment (112). However, there are alternative lines of evidence supporting that immune cells accumulating in the microenvironments and infiltrating into the tumor mass may have a role in eliminating cancer cells, as exemplified in ovarian caricinomas and melanomas (113,114). Tumors are tissue-specific diseases, and their invasive and metastatic phenotypes are highly diverse, as mentioned in the beginning of this review. We have discussed in this review that the majority of human ductal cancers may utilize the GEP100-Arf6-AMAP1 pathway for their malignancy that is activated by growth factor receptor signaling. Identification of the fine signaling pathways and mechanisms that are frequently used in each type of cancer for their invasion and metastasis will greatly contribute to the further understanding of the distinct outcomes of cancer-microenvironment interactions. Such information should then greatly contribute to the further development of cancer therapeutics, including immune therapy.
